# Effects of Content Support and Planning Instruction on Discourse Connection in EFL Argumentative Writing

**DOI:** 10.3389/fpsyg.2022.912311

**Published:** 2022-05-12

**Authors:** Yue Xie, Xiaoxuan Lv

**Affiliations:** ^1^School of Foreign Languages, Beihang University, Beijing, China; ^2^School of Foreign Languages, Beijing Forestry University, Beijing, China

**Keywords:** content support, planning instruction, discourse connection, argumentative writing, coherence, cohesion

## Abstract

Discourse connection is a challenging aspect of writing in a second language. This study seeks to investigate the effects of two classroom instructions on discourse connection in writing for EFL college students, focusing on their argumentative writing. Three classes were exposed to different pre-task conditions: receiving reading materials that provide content support for the writing, receiving planning instructions on effective outlining, and receiving no resources. The results showed that the instructions helped students attain better overall coherence in writing. However, noticeable differences between the two experimental groups emerged in terms of cohesion features. The reading group was found to employ more lexical cohesion devices in writing than the outline group, which indicated a heightened genre awareness. This inquiry helped us identify the reading group’s alignment with content support materials, particularly the change in stance as a factor that contributes to a higher level of lexical cohesion in writing.

## Introduction

The issue of discourse connection and a general lack of it in second language (L2) writing has received much scholarly attention (Lee, 2002; Todd et al., 2007; Plakans and Gebril, 2017). To improve discourse connection in L2 learners’ writing, researchers and instructors argue for classroom instructions that usually entails the teaching of connection strategies (e.g., Lee, 2002). However, studies along this line focus predominantly on the formal aspects of connection rather than the writer’s process of meaning making. To date, little research has investigated how supporting L2 writers’ cognitive basis may affect the quality of discourse connection. In addition, little is known about the relationship between context and meaning construction in a classroom setting (Yasuda, 2019).

To address the research gap, this study investigates the potential effect of two classroom instructions on discourse connection in learners’ writing. We chose to observe the instructional effects in argumentative writing, which is the most frequently adopted text type for assessing EFL learners’ academic writing proficiency (Zhang and Cheng, 2021; Zhang and Zhang, 2021). The classroom instructions we used are content support and planning instruction, which have been shown to be effective in lowering cognitive demands and improving the overall quality of writing (Abrams, 2019). However, their impacts on discourse connection remain to be determined. Since the instructions create different pre-task conditions, it may be necessary to explore the contextual effects on discourse connection through investigating whether the different instructions give rise to different connection features in the text.

## Literature Review

### Dimensions of Discourse Connection

Research on discourse connection in L2 writing has traditionally focused on two aspects: coherence and cohesion (Lee, 2002; Todd et al., 2007; Crossley et al., 2016; Plakans and Gebril, 2017). Coherence refers to the semantic and pragmatic relations between text segments that are perceived by a reader, whereas cohesion refers to the linguistic representations of such relations (Lee, 2002; Berzlánovich and Redeker, 2012; Plakans and Gebril, 2017). As a reader internal, process-oriented construct, coherence has been described mainly from a functional perspective as consistency in topic development (Todd et al., 2007) and evaluated holistically in writing assessment (e.g., Plakans and Gebril, 2017). As a text-based property, cohesion has been systematically categorized (Halliday and Hasan, 1976; Hoey, 1991; Tanskanen, 2006) and more objectively explored through manual or automated analysis (e.g., Crossley et al., 2016).

Research on cohesion in written discourse tends to concentrate on grammatical cohesion (i.e., the use of reference terms and conjunctions) and lexical cohesion (i.e., the connection between lexical items that are morphologically or semantically related). Studies show that these two categories of cohesion are the predominant means of discourse connection and display positive relations with writing quality (e.g., Reynolds, 2001; Liu and Braine, 2005).

Although models of cohesion and coherence represent the primary means to investigate discourse connection, they do not address the context of writing, nor do they satisfactorily explain the influence of context on text (Tanskanen, 2006; Berzlánovich and Redeker, 2012). Specifically, a text-centered analysis of discourse connection cannot account for the fact that lexical cohesion varies according to genre and writing conditions. As shown in L1 writing research, lexical cohesion is sensitive to register and genre (Taboada, 2004; Tanskanen, 2006; Blum-Kulka and Hamo, 2011; Fetzer, 2012). L1 expositions, in general, are found to be more cohesive than narrations (Hoey, 1991) and persuasions (Berzlánovich and Redeker, 2012). Similarly, in L2 research, texts that are more expository tend to rely more on the use of lexical devices for creating continuity (Guo et al., 2013; Llosa et al., 2020). Studies that compare the linguistic and discourse features of TOEFL-iBT writing show that integrated essays that connect writing with reading and listening are not only more formal but also more cohesive than independent essays (Guo et al., 2013; Riazi, 2016; Kim and Crossley, 2018). The findings regarding lexical cohesion imply that discourse connection should also be analyzed contextually, taking into account factors such as genre and writing conditions.

To explore the influence of context on discourse connection, genre awareness and its functional and linguistic manifestations in text need to be properly characterized. For this purpose, it is useful to consider the notion of stance. This is because stance is not only a catch-all feature in information-oriented argumentative writing (Hyland, 2005; Snow and Uccelli, 2009; Crosthwaite and Jiang, 2017) but also a major predictor of L2 writing quality at the tertiary level (Lee and Deakin, 2016; Crosthwaite and Jiang, 2017; Zhang and Zhang, 2021). According to Hyland (2005), stance is expressed through four linguistic features: hedges, self-mentions, boosters, and attitude markers. Among these four categories, hedges and self-mentions are the main stance markers in EFL expositions (McKinley, 2018). Hedges are markers of uncertainty that contribute to the cautious, objective stance in academic exposition (Xu, 2015; McKinley, 2018). Increased use of hedges is characteristic of academic exposition and indicates the writer’s accommodation of different points of view (Uccelli et al., 2013; Crosthwaite and Jiang, 2017; Qin and Uccelli, 2019). Self-mentions, according to Hyland (2005), include first-person pronouns and possessive adjectives that convey individual perspectives. Unlike hedges, reduced use of self-mentions aligns with the objective stance required for academic exposition (Shaw and Liu, 1998; Hinkel, 2004; Alfalagg, 2020). There is evidence that as pronoun use decreases, L2 writers’ expositions become more cohesive (Alfalagg, 2020).

In brief, a model of analysis for discourse connection should capture both the textual and contextual features of discourse. Observation of coherence and cohesion features is still fundamental to a text-centered analysis of discourse connection. Examination of contextual features such as stance markers may help us better understand how context influences learners’ awareness of genre expectation and the choice of suitable connection features.

### Instructions and Discourse Connection

Previous models of writing (e.g., Flower and Hayes, 1981; Abdel Latif, 2021) inform our choice of instructional activities. These models show that two resources—topic knowledge and writing plans—are essential for effective writing. In L1 writing research, it has been demonstrated that extensive topic knowledge results in higher-quality texts (Spivey, 1990; Proske and Kapp, 2013). In L2 studies, however, topic knowledge has often been treated as a constant or examined only in association with the language aspects of writing (e.g., Shi and He, 2012; Yang and Kim, 2018). There is little inquiry into how increased topic knowledge influences discourse connection. For the other cognitive resource, planning helps a writer explore the connections between ideas. In EFL writing classroom, it is a common practice to use outlines to plan for an essay (Bai et al., 2014, 2020; Chen and Ren, 2021). However, the cognitive benefit of outlining on the discourse aspects of writing is yet to be established. Unlike L1 studies, which generally find a positive influence of outlining (e.g., Kellogg, 1988; Galbraith et al., 2005), L2 studies show that outlining may exert no or even a negative impact on writing quality (Ong and Zhang, 2010; Johnson et al., 2012). An explanation for such divergent results is that L2 writers in general do not make as effective plans in the outlining condition as L1 writers (Johnson et al., 2012; Khezrlou, 2020). To ensure the effective use of outlines in essay planning, formal instruction on the principles of outlining may be necessary.

Although content support and planning instruction may strengthen EFL writers’ cognitive base for writing and promote discourse connection, direct evidence of such relations is limited in the literature. Most studies on these two types of instructions are concerned with linguistic performance rather than the discourse aspects of writing.

#### Content Support and Discourse Connection

There is evidence to show that content support can lead to conceptual and linguistic alignment between the input materials and the learner’s written output (e.g., Wang and Wang, 2015; Zhang and Zhang, 2021). For instance, Crossley et al. (2013) found that L2 writers were likely to replicate or imitate linguistic forms and structures from the task prompt. However, research on content support has mainly investigated its influence on the fluency and complexity of L2 writing (e.g., Ong and Zhang, 2013; Révész et al., 2017; Abrams, 2019; Jung, 2020). Despite a linguistic focus, these studies revealed that both the content and organization scores for writing improved when content support was available. Similarly, in recent studies on story continuation tasks, access to the original story was shown to have a positive influence on writing quality (Wang and Wang, 2015; Peng et al., 2020; Zhang and Zhang, 2021). However, there has been little empirical research on the connection between content support and coherence in L2 writing.

Findings relating to cohesion in content support conditions mainly come from research that compares integrated essay responses with independent essay responses in TOEFL-iBT (Guo et al., 2013; Riazi, 2016; Kim and Crossley, 2018). It was found that the density of lexical cohesion correlated with integrated essay scores but not with independent essay scores. Moreover, integrated essays were shown to be more cohesive than independent essays. In independent essays, writers tend to argue through personal opinions and life experiences (Guo et al., 2013). In integrated essays, writers generally assume an objective stance, which leads to more information-centered texts with high-cohesion density (Guo et al., 2013; Llosa et al., 2020).

#### Planning Instruction and Discourse Connection

Most studies that explore the relationship between planning and L2 writing quality have mainly focused on the linguistic dimensions of writing (e.g., Johnson et al., 2012; Ong and Zhang, 2013). However, the results from these studies suggest that planning has a positive influence on overall writing in terms of content and text organization (Rahimi and Zhang, 2017). There is but one instructional study that directly examined the influence of instructed planning on coherent expression. Chang et al. (2020) compared the impact of two instructional approaches: using a concept map for organizational planning and using theme–rheme patterns to structure topic information. While improvement in discourse management was observed for both instructional groups, the group that received planning instruction displayed better control over discourse connection at the posttest.

Very few studies have explored how instructed planning affects the cohesion of EFL writing. However, Zhang (2018) discovered that the planning group significantly outperformed the control group on measures of cohesion between sentences and between paragraphs. These findings, plus student interviews, suggest that instructed planning could channel EFL writers’ attention to the role of cohesion in text organization. This awareness was transferred to the composing process and resulted in increased connectivity in writing.

The works, cited above, offer support for using cognitive, meaning-focused instructions to improve discourse connection in EFL argumentative writing. However, some limitations must be noted. First, most of the studies only lend indirect support to the impact that content support and instructed planning may have on discourse connection. This is because a principal concern in these investigations is L2 writers’ linguistic performance, not their ability to manage discourse. Therefore, the instructional effects on coherence and cohesion, the two fundamental aspects of discourse connection, have not been systematically measured. Next, the existing literature focuses primarily on the product of writing (Lv et al., 2021), with little regard for the process of writing. Although a high level of lexical cohesion has been found to co-occur with the academic stance in exposition and integrated writing, this interface remains under-investigated in EFL writing research. In summary, more evidence is needed to demonstrate how content support and planning instruction may facilitate discourse connection in EFL argumentative writing. In the present study, we chose to explore two dimensions of discourse connection in student writing: coherence (based on perceived meaning connections in a text) and cohesion (based on formal connection features in a text). Specifically, the study seeks to investigate the following two research questions:

1. To what extent do content support and planning instruction influence coherence in EFL learners’ argumentative writing?
2. To what extent do content support and planning instruction influence cohesion in EFL learners’ argumentative writing?

## Methods

### Participants

The participants are Chinese EFL learners from a research university in China. They belonged to three class cohorts in an EAP course program. Prior to course registration, they attended a placement test of general English proficiency and they were considered to represent upper-intermediate to advanced English learners in terms of general proficiency. Although data relating to individual writing proficiency was unavailable, variance analysis of their test scores showed little or even negligible differences between the three cohorts. The three classes of participants were randomly assigned to the three pre-task conditions that the present study aimed to explore. As a result, there were 31 participants in the reading group, 29 in the outline group, and 34 in the control group. Their ages ranged from 16 to 19 and they all majored in science. The gender ratio in these three groups was roughly 5 males to 1 female.

As the participants attended the same EAP program, they were roughly on an equal footing in terms of exposure to academic English. The use of a common syllabus helped to guarantee a relatively high degree of uniformity between classes in the choice of learning materials and instructional activities. As the instructions were carried out during regular class time, each intact class was randomly assigned to one of the following three groups: the reading group that received content support in the form of printed passages, the outline group that received instruction on how to plan in the outline form and the control group.

### Research Design

The study followed a quasi-experimental design with a pretest, treatment, and a posttest. The pretest was used to ensure intergroup homogeneity before treatment, and the posttest was meant to show the treatment effects. Two similar versions of timed impromptu writing tests (see Appendix **A**) were developed after consulting four experienced teachers. They are similar in that they can elicit an argument that is developed through comparison and contrast.

After piloting the tests with 12 students from a comparable population, it was determined that 50 min were needed to complete each essay. The tests were computer-delivered and conducted online for the sake of timekeeping. Participants were asked not to consult a dictionary or any other reference materials during the test, whether online or offline. No comments on the writing were given during the week between the two tests.

### Treatment Materials

Two passages (Passages 1 and 2) that were thematically connected were chosen as input materials for the reading group. These two passages complement each other by presenting contrasting facts and statistics that highlight the differences between men and women. The two passages were piloted on 12 students from a similar but separate cohort who reported no difficulty with the content or the language.

For the reading group, the reading handout was distributed at the beginning of a session and taken back when the session was over. Participants were encouraged to read the passage at their own paces and were required to finish the accompanying questions. The teacher did not provide comprehension support except for checking answers at the end of each session.

For the outline group, we designed two instructional sessions that featured explicit teaching on the use of an outline to plan for an essay. Each session contained a variety of instructional activities, including (1) watching two videos in the format of an outline, (2) discussing an exemplary outline, (3) drafting outlines for an essay prompt, and (4) evaluating the outline drafts with peers. These activities were meant to raise students’ awareness of text organization by imparting the knowledge of outline structure.

### Procedure

The two writing tests were administered one week apart, and the posttest was conducted on the treatment day. Participants from the experimental groups attended two separate sessions of treatment. Each session was 20 min in length, with a 50-min interval in between for learning activities specified on the course syllabus. The reading group was instructed to finish Passage 1 on a handout that was collected after 20 min. This procedure was repeated when Passage 2 was used. Similarly, for each training session, the outline group received instruction handouts which were taken back at the end. To ensure comparability between groups in time allotment, the control group also participated in two 20-min sessions of in-class reading on materials that were unrelated to the test topic.

### Data Analysis

To discriminate coherence from other aspects of writing, coherence in this study was scored separately from content and language. A primary-trait rating rubric, based on Bamberg (1984), was developed to describe coherence features at the paragraph level (see Appendix **B**). This rubric differs from previous coherence scales by disentangling coherence in body paragraphs from coherence at the passage level. We chose to focus on paragraph-level coherence because studies have shown that problems within body paragraphs constitute a major source of breakdowns in coherent interpretation (Knoch, 2007; Wang et al., 2012). Paragraph coherence was assessed for three features: (1) there is one and only one main idea for a paragraph, (2) there is a discernible pattern of development, and (3) there is a clear topic sentence. These features were then transformed into a five-point scale. In addition, a 0.5 decimal point was suggested to the raters so that they could easily determine the score for an essay that did not meet all descriptors for a scale level. All pretest and posttest essays were double-marked by two EFL teachers, who have extensive training and experience in rating writing performance tests. The Pearson correlation coefficient of the two raters’ scores ranged from 0.82 to 0.91 for all subsets of essays grouped according to tests and treatments. The final scores for coherence were the averages of the two raters’ scores.

The scoring of cohesion is an objective procedure that involves counting the explicit linguistic markers of cohesion. We used Coh-Metrix 3.0 to measure four groups of variables that “reflect certain cognitive operations underlying coherence judgment” (Crossley et al., 2008: 490) and are frequently adopted in cohesion studies (e.g., Aryadoust and Liu, 2015; Crossley et al., 2016; Hou, 2017). These four variable groups are Referential cohesion, LSA (latent semantic analysis), Connectives, and Lexical diversity (see Table 1).

**Table 1:** Cohesion measures and corresponding indices (adapted from McNamara et al., 2014).

Category	Feature	Indices	Description
Referential Cohesion	Noun overlap	CRFNO1/a	Same form repetition of a common noun, between adjacent sentences/all sentences
	Argument overlap	CRFAO1/a	Noun overlap in singular or plural forms, between adjacent sentences/all sentences
	Stem overlap	CRFSO1/a	Overlap between nouns and words of shared stems, for adjacent sentences/all sentences
	Content word overlap	CRFCWO1	Shared content words between adjacent sentences, including pronouns
	anaphor overlap	CRFANP1	Overlap between a pronoun and another pronoun or noun in the earlier sentence
LSA	LSA sentence adjacent	LSASS1	Mean of LSA cosines for adjacent sentences
	LSA sentence all	LSASSp	Mean of LSA cosines for all sentence pairs
	LSA paragraph	LSAPP1	Mean of LSA cosines between paragraphs
	Givenness	LSAGN	Average givenness of each sentence
Connectives	All connectives	CNCAll	The incidence of all connectives
Lexical Diversity	Lexical diversity	LDMTLDa	The range of vocabulary in text

Referential cohesion refers to lexical coreference or content word overlap beyond the boundary of a sentence. Measures in this category are calculated in terms of the frequency of exact repetitions and morphologically related words. LSA captures deeper-level connectivity that is calculated on basis of the semantic relatedness of the words in adjacent or adjoining sentences. Studies show that LSA values can discriminate between L1 and L2 writings (Crossley and McNamara, 2009) and between L2 writing of different qualities (Green, 2012). Connectives are transitions that reveal the logical connection between sentences. Coh-Metrix calculates 9 indices of connectives in 5 classes: causal (because, so), logical (and, or), temporal (first, then), additive (and, moreover), and adversative (whereas, although). Lexical diversity refers to the variety of unique words in a text. More unique words generally indicate higher lexical sophistication. However, lexical diversity also affects lexical repetition which is a major mechanism for creating cohesion.

## Results

### Effects on Coherence

To examine the treatment effects, the holistic scores for coherence were compared between groups and across time (see Table 2). A one-way ANOVA was performed on pretest scores to establish the comparability of groups, and no differences were found (*F* (2, 91) = 2.006, *p* = 0.117, η2 = 0.053). As the differences between groups enlarged in the posttest, another one-way ANOVA was conducted on posttest scores, but the result was still insignificant (F (2, 91) = 1.519, *p* = 0.213, η2 = 0.21).

**Table 2:** Descriptive statistics for coherence score by group and session.

Group	*n*	Pretest Mean (SD)	Posttest Mean (SD)
Reading	31	3.85 (0.22)	3.94 (0.18)
Outline	29	3.82 (0.23)	3.92 (0.28)
Control	34	3.84 (0.22)	3.89 (0.20)

*Maximum coherence score = 5.*

### Effects on Cohesion

Paired t-tests were performed to explore the direction as well as the degree of change in selected Coh-Matrix variables. Table 3 shows that between the two tests, the level of referential cohesion (i.e., CRFNO1/a, CRFAO1/a, CRFSO1/a, and CRFCWO1) did not change much, but the LSA measures changed dramatically. However, the reading group did not display a significant drop in LSASS1 value at the posttest, which suggests a relatively higher level of connection between adjacent sentences. In contrast, the outline group showed a radical drop in all LSA measures, which suggests less shared semantic information between sentences. Moreover, the outline group displayed a significant increase in connective use at the posttest (CNCAll, t = 2.99, *p* < 0.01). Taken together, the findings on the outline group indicate a tendency to connect text more through grammatical cohesion than through lexical cohesion.

**Table 3:** Results of paired t-test for cohesion scores.

Variables	Reading Group			Outline Group			Control Group		
	M	SD	t	M	SD	t	M	SD	t
CRFNO1	−0.03	0.35	−0.42	−0.07	0.41	−0.92	0.04	0.33	0.65
CRFAO1	0.02	0.29	0.32	−0.06	0.32	−1.00	0.01	0.31	0.21
CRFSO1	−0.01	0.29	−0.12	−0.03	0.41	−0.36	0.03	0.31	0.60
CRFNOa	0.01	0.33	0.09	−0.08	0.35	−1.22	−0.01	0.33	−0.14
CRFAOa	0.05	0.26	1.18	−0.10	0.27	−1.92	0.00	0.28	−0.04
CRFSOa	0.04	0.28	0.89	−0.06	0.34	−0.87	0.00	0.31	0.05
CRFCWO1	−0.01	0.08	−0.41	−0.03	0.07	−1.99	−0.02	0.08	−1.56
CRFANP1	−0.13	0.23	−3.21^**^	−0.09	0.30	−1.58	−0.18	0.29	−3.59^**^
LSASS1	−0.04	0.11	−1.90	−0.10	0.12	−4.5^***^	−0.05	0.09	−3.37^**^
LSASSp	−0.04	0.10	−2.13^*^	−0.10	0.11	−5.0^***^	−0.05	0.10	−2.97^**^
LSAPP1	−0.13	0.20	−3.7^***^	−0.15	0.28	−2.97^**^	−0.13	0.20	−3.8^***^
LSAGN	−0.03	0.06	−2.35^*^	−0.05	0.07	−4.1^***^	−0.04	0.05	−4.1^***^
LDMTLDa	2.14	17.71	0.67	5.86	22.05	1.43	12.35	28.78	2.5^*^
CNCAll	4.37	28.15	0.86	15.13	27.28	2.99^**^	5.99	30.89	1.13

* *Difference is significant at the 0.05 level (2-tailed).*

** *at the 0.01 level (2-tailed)*.

*** *at the 0.001 level (2-tailed)*.

Next, a one-way MANOVA was performed to determine whether significant differences existed between groups at the posttest. Using Pillai’s trace, the MANOVA revealed a significant difference (V = 0.62, F (2, 91) = 1.37, *p* = 0.04, partial η =0.21), and the Levene test showed no violation of the assumption of homogeneity of variation. Four Coh-Metrix variables displayed significant differences between pairs of groups (see Table 4).

**Table 4:** Results for significant differences between groups at posttest.

Coh-Metrix indices	Group	Mean	SD	Levene test (*p)*	F	*p*	*η*2	Significant Pairwise Differences (p)
CRFCWO1	Reading	0.14	0.05	0.06	5.20	0.002	0.113	G1 > G2(0.00)
	Outline	0.10	0.03					G1 > G3(0.01)
	Control	0.11	0.04					
LSASS1	Reading	0.20	0.07	0.17	5.64	0.001	0.121	G1 > G2(0.00)
	Outline	0.16	0.05					G3 > G2(0.01)
	Control	0.19	0.06					
LSASSp	Reading	0.20	0.06	0.65	4.38	0.006	0.097	G1 > G2(0.00)
	Outline	0.15	0.06					G3 > G2(0.01)
	Control	0.20	0.06					
LDMTLDa	Reading	70.57	17.26	0.48	7.12	0.000	0.148	G2 > G1(0.00)
	Outline	95.08	21.85					G3 > G1(0.00)
	Control	88.59	24.94					

*p (Fisher’s LSD): the difference is significant at the 0.05 level (2-tailed). G1 = reading group, G2 = outline group, G3 = control group.*

First, the difference in the measure of content word overlap (CRFCWO1) reached the level of significance (F (2, 91) = 5.20, *p* = 0.002, partial η =0.11). The post-hoc tests confirmed that the reading group had a significantly higher value for CRFCWO1. For LSA between adjacent sentences (LSASS1), the difference between groups was also significant (F (2, 91) = 5.64, *p* = 0.001, partial η =0.12). The post-hoc comparisons revealed a significantly higher LSASS1 for the reading group and the control group in comparison with the outline group. Next, LSA between paragraphs (LSASSp) was another discriminating factor, and a significant difference existed between the outline group and the reading group. For lexical diversity (LDMTLDa), significant differences were found between the reading group and the other two groups (F (2, 91) = 7.12, *p* = 0.000, partial η =0.15).

In sum, the results of the between-group comparison highlighted the difference in cohesion between the reading group and the outline group. At the posttest, the significant results indicated an enlarged gap between the reading group and the other two groups in terms of using explicit content words to connect sentences. In contrast, the outline group relied on grammatical means to achieve connectivity.

## Discussion

To gauge the impact of content support and planning instructions on discourse connection, we performed multiple analyses on the post-instruction writings and compared cohesion and coherence measures. The results of the holistic rating show that both instructions could elevate the quality of coherence. More importantly, findings related to lexical cohesion and stance features suggest that the two instructions shaped meaning making in different ways.

### Effects of Pedagogical Support on Discourse Connection

The research questions focus on the impact of instructions on the textual aspects of discourse connection. As far as the coherence score is concerned, the reading group has the best performance at the posttest. This finding is in accord with previous studies that show a positive influence of content support on the quality of argumentative essays (Ong and Zhang, 2013; Révész et al., 2017; Abrams, 2019). Lending further support to the effectiveness of content support is the finding that this method induced high-cohesion texts. As shown in Table 4, the reading group’s posttest essays are more lexically connected for adjacent sentences (i.e., CRFCWO1 and LSASS1) and between paragraphs (LSASSp). These results align with studies that compare lexical features in writing when content support is or is not available (Guo et al., 2013; Riazi, 2016; Kim and Crossley, 2018). For instance, Riazi (2016) indicated that the integrated essays of TOEFL-iBT displayed higher values for the Coh-Metrix measures of content word overlap (CRFCWO1) and semantic similarity (LSASS1).

A positive influence on discourse connection was also found for the planning instructions. As the outline group outperformed the control group in coherence score, the instructions proved to be effective in inducing better overall coherence. This finding substantiates the cognitive benefits of pre-task planning in second language writing (Rahimi and Zhang, 2017) and is also in line with the study of Chang et al.’s (2020), which reveals guided planning’s positive influence on discourse connection.

However, the pattern of cohesion in outline group essays does not corroborate the study of Zhang (2018), who reported an increase in the Coh-Metrix measures of referential cohesion (i.e., CRFCWO1 and CRFAO1). Compared to the other two groups, the outline group’s writings display a lower level of referential cohesion (CRFCWO1) and substantially lower level of semantic associations (LSASS1 and LSASSp). This means that unlike the participants in Zhang’s study (2018), the outline group in the current investigation did not resort so much to lexical repetition in writing. The discrepancy in findings may lie in the duration of the planning instructions. Zhang’s participants received four training sessions over two months and were given ample time to reflect at each session, whereas the outline group was exposed to just 40 min of instruction. As growth in the use of lexical cohesion was mainly reported by longitudinal studies (Crossley et al., 2016; Hou, 2017), the low level of lexical cohesion in outline group writings was not entirely unexpected.

### Different Effects on Cohesion between the Two Instructions

The current study shows that content support fosters coherent expression. Essays produced by the reading group prove to be both more coherent and more cohesive. In contrast, instructions on outline give rise to more coherent but less cohesive writing. A low level of lexical cohesion in outline group essays was confirmed by statistical analysis (see Table 4). If the outline group relied less on lexical cohesion but still managed discourse connection, other mechanisms for establishing coherence should have been in place.

The paired t-test for Coh-Metrix indices helped identify two important cohesive devices in outline group essays. First, as shown in Table 3, outline group writers used substantially more logic connectives in the posttest (CNCAll: t = 2.99, *p* < 0.01), which indicates an increased tendency to explicate the logical connections between text segments. Therefore, it might be safe to conclude that the outline group preferred to use cohesive devices for creating connectivity.

A follow-up question to ask is why different degrees of cohesion and different choices of cohesive devices are observed for the two instructional groups. It is necessary to bear in mind that a high degree of cohesion has generally been observed for academic exposition (Biber, 1995; McGee, 2009; Berzlanovich and Redeker, 2012). In this sense, the reading group’s dense lexical cohesion and objective stance are characteristics that align with the genre of exposition. Moreover, a shift from less cohesive and subjective writing to more cohesive and impersonal writing is generally considered to be a sign of development in L2 learners’ formal language ability (Shaw and Liu, 1998; Choi and Kim, 1999; Hinkel, 2004). For instance, Choi and Kim (1999) found that advanced EFL students created adequate lexical cohesion for sentence-level connection, whereas lower-level students often failed to produce enough lexical cues between adjacent sentences. Some longitudinal studies also found that over time, the argumentative essays of EFL college students improved significantly in several Coh-Metrix indices that measure the degree of lexical cohesion (Aryadoust, 2016; Crossley et al., 2016; Hou, 2017).

### Implications

This study implies that research related to discourse connection needs to consider various factors that characterize language use. The need to study discourse connection from multiple perspectives is highlighted by the fact that the two experimental groups were rated similarly in terms of overall coherence but displayed significantly different degrees of cohesion in writing. To explore the extent of impact that different instructions have on discourse connection, we employed Coh-Metrix analysis to uncover hidden textual features and found that only content support resulted in a higher level of cohesion in writing. However, to describe the nature of the impact on writing, it is necessary to dig deeper into lexical features during the process of meaning making. In brief, the present study echoes previous research (Lee, 2002; Hou, 2017) and further demonstrates the necessity of using mixed methods to research EFL or ESL writing (ref. Zhang and Cheng, 2021).

The findings of the study also have some pedagogical implications for EFL teachers and writing instructors in the EFL context. First, as both types of instructions were shown to contribute to coherence in writing, it is advisable to use them more frequently in EFL writing classrooms. In addition to fostering coherent expression, content support can enrich student writers’ knowledge schema and provide fodder in the form of linguistic structures (Zhang, 2017; Abrams, 2019). Planning instruction or guided planning in general helps to draw out learners’ creative potential and provides essential knowledge about discourse organization that is transferable to task performance. Therefore, a combination of both instructions would help EFL learners produce better organized and more sufficiently developed writing. Second, as high-cohesion density was found for reading-based writing, it might be worthwhile to further exploit this or similar approaches as ways to orient EFL learners to the pragmatic norms of formal exposition. This idea echoes the pragmatic-based view of language instruction proposed by Snow and Uccelli (2009), which considers not only the teaching of language forms but also ways to foster academic language ability and sociocognitive development. Although lexical cohesion is crucial for creating discourse connection in exposition, it is difficult to teach in the EFL context (Mahlberg, 2009). Likewise, the objective stance expected for formal exposition is a challenging element in EFL writing pedagogy (Xu, 2015). Given the reading group’s gains in these aspects of writing, content provision may be an effective approach to teaching argumentative writing (Wang and Wang, 2015; Ye and Ren, 2019).

## Conclusion

This study explored how instructions that tapped into EFL learners’ cognitive resources may affect discourse connection in argumentative writing. Pedagogical support in the current investigation was operationalized as topic-related reading input and instructions on how to plan a text in outline form. The results of coherence scoring showed that both instructions enabled EFL learners to express themselves more coherently. However, the results of cohesion scoring revealed the two instructional groups’ differentiated patterns of lexical cohesion in writing. Compared with the outline group, the reading group’s essays display a significantly higher level of cohesion, and the discourse connection is particularly strong for adjacent sentences. It might be argued that the increased use of lexical cohesion facilitates topic development in the argumentative text.

The findings of the current study, however, should be interpreted in light of some limitations. First, as with most empirical studies on L2 writing coherence, the individual variations in both the process and the products of writing cannot be fully controlled. Despite the emerging group trend, variations exist from one text to another and even from one paragraph to the next in the same essay. The generalizations made for a group by no means describe accurately an essay within the group. Furthermore, as learners’ cognitive and affective differences could result in varied degrees of uptake of the instructional support, it might be necessary to modify the instructional approaches to better meet learners’ needs (ref. Liu and Chu, 2022). A second limitation is that the tools for investigating discourse connection were chosen primarily to elucidate lexical cohesion. Despite being a significant source of coherence, lexical cohesion alone is insufficient for producing a coherent text. Last, the current study is also limited in studying only the linguistic manifestations of discourse connection and the short-term effects of instructions. No information was gathered about the process of writing to understand the critical choices learners made to maintain text unity and improve surface connectivity. Therefore, future research could consider the use of verbal reports (e.g., Ren, 2014) to collect process data for constructing a coherent English argumentative essay and use a longitudinal design to corroborate the findings of this study.

## Data Availability Statement

The original contributions presented in the study are included in the article/Supplementary Material, further inquiries can be directed to the corresponding author.

## Author Contributions

YX was responsible for conceptualization and methodology design, participated in data analysis, and prepared the original draft. XL carried out data analysis, and was also responsible for the correction and revision of the manuscript. All authors contributed to the article and approved the submitted version.

## Conflict of Interest

The authors declare that the research was conducted in the absence of any commercial or financial relationships that could be construed as a potential conflict of interest.

## Publisher’s Note

All claims expressed in this article are solely those of the authors and do not necessarily represent those of their affiliated organizations, or those of the publisher, the editors and the reviewers. Any product that may be evaluated in this article, or claim that may be made by its manufacturer, is not guaranteed or endorsed by the publisher.

## Appendix A

Pretest prompt.

Two views concerning university’s attendance policy are given below:

It would be in the students’ best interests if optional attendance is in place.

It would be in the students’ best interests if mandatory attendance is in place.

In writing your composition, discuss which of the above views is closer to your own. Use specific reasons and examples to support your view.

Posttest prompt.

Two different views concerning gender differences are given below:

Although men have many advantages in life, it is easier to be a woman.

Although women have many advantages in life, it is easier to be a man.

In your writing, answer the question “Is it better to be a man or a woman?” by choosing one view above that is closer to your own. Use specific reasons and examples to support your view.

## Appendix B

**Table:** Rating rubric for coherence with level descriptions.

Level	Coherence
5	1) Each paragraph has one main topic, and all content materials are highly relevant to the main topic; 2) Each paragraph is clearly organized and adequately develops the main topic; 3) Each paragraph has a topic sentence at the beginning and the topic sentence summarizes all following sentences.
4	1) Each paragraph has one main topic, but there are minor digressions from the main topic; 2) Each paragraph is organized and develops the main topic; 3) Each paragraph has a topic sentence at the beginning, but the topic sentence does not summarize all following sentences.
3	1) Each paragraph has one main topic, but there are serious digressions from the main topic; 2) The paragraph has no adequate organizational plan but relies on the listing of content materials; 3) The topic sentence is missing from a paragraph and/or does not summarize all following sentences.
2	1) There is a barely identifiable main topic for each paragraph; 2) The paragraph has minimal organizational plan and basically relies on the listing of content materials; 3) No topic sentence can be identified.
1	1) The topic of a paragraph cannot be identified; 2) The paragraph has no organizational plan.

*An example of inadequate topic sentence.*

*Besides, it’s easier for men to be independent both economically and politically. As we know, men are thought to be the significant roles in family. In some degree, men play a vital role in the family’s choice and development. In other words, it’s up to men for the family to head for future. Women are mainly thought to be moms to raise babies, do housework, and help their husbands. Their lives seem to be linked to their family, so that they have too many things that needing to be handled to have enough freedom. Or, they rely on their husbands, their children, and their family. Sometimes, they cannot follow their hearts to make their own choices for the interest of the whole family*.

*The first sentence of this paragraph builds up the anticipation for evidence that supports men’s financial independence and engagement in the social and political life. However, the subsequent sentences discuss the different roles between men and women in family situations, highlighting how men are less restrained by domestic responsibilities. The first sentence in the paragraph above just gives the impression of a topic sentence but fails to subsume the subsequent sentences*.
